# Expression and importance of matrix metalloproteinase 2 and 9 (MMP-2 and -9) in human trophoblast invasion

**DOI:** 10.1186/1477-7827-2-59

**Published:** 2004-08-04

**Authors:** Elsebeth Staun-Ram, Shlomit Goldman, Diana Gabarin, Eliezer Shalev

**Affiliations:** 1 Laboratory for Research in Reproductive Sciences, Department of Obstetrics and Gynecology, Ha'Emek Medical Center, 18101, Afula, Israel; 2 Rappaport Faculty of Medicine, Technion-Israel Institute of Technology, Haifa, Israel

## Abstract

**Background:**

The aim of this study was to examine the invasiveness of first trimester trophoblasts according to the secretion profile of MMP-2 and -9 at different gestational stages, and to test the similarity between primary trophoblast cell-culture and the JAR choriocarcinoma cell-line.

**Methods:**

First trimester trophoblasts were divided into two groups: 6–8 weeks (early) and 9–12 w (late) of gestation. The two trophoblast groups and JAR cells were cultured in medium, with various concentrations of forskolin and Epidermal Growth Factor (EGF). Proteolytic activity was detected by zymography and invasiveness was assessed by Matrigel invasion assay. Student's T-test was used for statistical analysis.

**Results:**

In 6–8 w trophoblast, proMMP-2 was only slightly dominant over proMMP-9 (53.2% vs. 46.8% respectively), whereas in 9–12 w, proMMP-9 was clearly dominant over proMMP-2 (61.7% vs.38.3% respectively). In JAR cells proMMP-2 was strongly dominant (90.2% vs.9.8% respectively). In JAR cells forskolin significantly increased proMMP-2 and -9 secretion (128.5% +/- 12 and 183.2% +/- 27.9 of control, respectively). EGF had a dual effect on JAR cells: at 8 ng/ml both proMMP-2 and -9 were increased (133.5% +/-15 and 223.9% +/- 32.4 of control, respectively) while at 80 ng/ml both proMMP-2 and -9 were decreased (65.1% +/- 18.3 and 66.6% +/- 37 of control, respectively). Forskolin significantly increased both proMMP-2 and -9 secretion in 6–8 w and 9–12 w trophoblasts (125.9% +/- 6.3,128.4% +/- 6.4; 169.7% +/- 20.3, 120.3% +/- 4.5 of control, respectively). EGF also significantly increased both proMMP-2 and -9 secretion in 6–8 w and 9–12 w trophoblasts (141.22% +/- 14.8, 138.8% +/- 10.3; 168.3% +/- 18.2, 117.3 +/- 3.8 of control, respectively). Both forskolin and EGF increased trophoblast cells invasiveness in all groups. The invasive ability of trophoblast cells, induced by forskolin, was reduced by MMP-2 antibody in: JAR cells, 6–8 w and 9–12 w trophoblasts. Likewise trophoblast invasion induced by EGF was reduced by MMP-2 antibody in all groups. However the invasive ability induced by forskolin or EGF was inhibited by MMP-9 antibody only in trophoblasts from 9–12 w.

**Conclusions:**

First trimester trophoblasts express differential gelatinase secretion profile according to the gestational week. In JAR and early trophoblasts (6–8 w) MMP-2 is the main gelatinase and the key enzyme in trophoblast invasion. Thereafter in late first trimester trophoblasts (9–12 w), both MMP-2 and -9 participate in trophoblast invasion.

## Background

Successful implantation depends on the ability of the embryo to degrade the basement membrane of the uterine epithelium and to invade the uterine stroma. Cytotrophoblastic cells (CTB) are derived from trophoectodermal cells of the blastocyst. CTB ensue to become either the villous cytotrophoblastic cells which will proliferate and differentiate by fusion to form the syncytiotrophoblast, or they will stream out of the syncytiotrophoblast to form mononuclear multilayered invasive extravillous cytotrophoblastic cells. The temporal and spatial regulation of trophoblast invasion is mediated in an auto-and paracrinic way by trophoblastic and uterine factors [1]. Several factors have been studied, including hormones, cytokines and growth factors [2,3]. Trophoblast invasion is facilitated by degradation of the extracellular matrix of the endometrium/decidua by various proteinases, among them, the matrix metalloproteinases (MMPs). The tissue inhibitors of matrix metalloproteinases (TIMPs) inhibit the activity of the MMPs by binding to the highly conserved zinc-binding site of active MMP [4]. Successful implantation and trophoblast invasion are closely linked to the expression of MMPs, which are able to degrade basement membranes. The gelatinases (gelatinase A: MMP-2: 72-kDa and gelatinase B: MMP-9: 92-kDa) which degrade collagen IV, the main component of the basement membrane, are expressed by trophoblast cells and are therefore regarded as key enzymes in the invasion process [5]. Several studies have shown that MMP-2 and MMP-9 synthesis and activation are required for trophoblast invasion [1,5-9]. Some studies have found either MMP-9 [5,7,8], or MMP-2 [9-11] to be more pronounced during the first stage of trophoblast invasion. However, the exact changes in protease expression during the first trimester are still not clear. Xu et al [11] found differential expression of MMP-2 and -9 in first trimester trophoblast cells, with MMP-2 being the main gelatinase secreted until 9 w and hereafter MMP-9. In this study, trophoblast cells from first trimester were therefore divided into two groups according to their MMP secretion profile. The aim of this study was to examine the expression and importance of MMP-2 and -9 in human trophoblast invasion, and to test the similarity between primary trophoblast cell culture and the JAR choriocarcinoma cell-line. The JAR cell-line serves as a widely used model for 1^st ^trimester trophoblasts [12-14]. The limited availability of 1^st ^trimester trophoblast tissue often requires the use of such a model, and therefore a comparison study between JAR cell-line and 1^st ^trimester trophoblasts is of significant importance to ensure similarity. This study shows a differential, dynamic importance of each gelatinase in trophoblast invasion during the 1^st ^trimester.

## Methods

### Cell culture

The JAR (Jar, HTB 144) human choriocarcinoma line was established from a trophoblast tumor of the placenta (1988 American Type Culture Collection Catalogue). The JAR cells were a generous gift from Prof. Hochberg A Department of Biological Chemistry, Hebrew University, Jerusalem, Israel. JAR cells (1 × 10^4 ^cells/well) were cultured in M-199 medium (Beit-Ha'Emek, Israel) containing 10% Fetal Calf Serum (FCS, Beit-Ha'Emek, Israel) and penicillin/streptomycin (Beit-Ha'Emek, Israel). Cell culture was maintained in a humidified atmosphere containing 5% CO_2 _at 37°C. After 24 hours of culture to facilitate cell attachment, the medium was removed, and M-199 medium with 1.5% serum supplemented with antibiotics was added. The cells were cultured with various concentrations of: a) Forskolin 1–100 μM, b) Epidermal Growth Factor (EGF), 0.8–80 ng/ml (Sigma). Control consisted of M-199 with 1.5% serum alone. Cells were cultured for an additional 48 hours, and media were removed for analysis of MMP secretion and stored at -20°C until use. Cell count was performed with XTT in order to normalize MMP secretion to cell number.

### Isolation and cultivation of human cytotrophoblast cells

**Figure 1:**
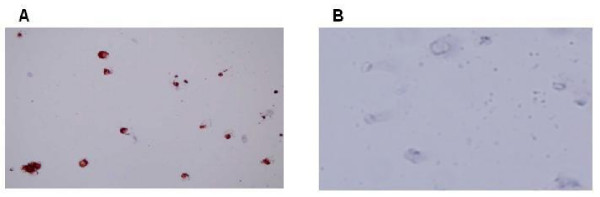
Representative immunohistochemical analysis of isolated placental cells (6–8 w) after 24 h in culture. (A) Cells stain positive for anti-human cytokeratin 7 (1:100, Biogenics). (B) Cells stain negative for anti-human vimentin (1:200, Zymed). Magnification: ×100.

Human trophoblast cells were obtained from legal abortions (6 to 12 weeks of gestational age), with the approval of the local ethical committee (in compliance with the Helsinki Declaration) and the consent of the participating patients. Trophoblast cells were isolated as described previously in detail elsewhere [8,9,15,16] with modifications. Briefly, tissues were digested by 0.25% trypsin (Sigma) and DNase I (Sigma) at 37°C, then trophoblast cells were separated from blood cells and decidua on a discontinuous Percoll gradient (Sigma) and immunopurified with magnetic antibody CD45RB (DAKO, Denmark). The cells were plated at 1–2 × 10^5 ^cells/well in 96-well plates or in Transwell plates (Corning) with M-199 medium supplemented with 1.5% FCS and 1% penicillin/streptomycin and kept in 5% CO2 at 37°C. Inducers (10 μM forskolin or 8 ng/ml EGF (chosen as working concentrations after a dose-response study in JAR cell-line) were added to medium, and after 48–72 h media were collected for analysis of MMP secretion and cell count was performed. This method supplies a 95–98% purity of trophoblastic cells, including all trophoblastic sub-groups. We verified the purity of trophoblast cells by using immunohistochemistry with specific antibodies to cytokeratin 7 (positive) and vimentin (negative), commonly used for indication of trophoblast purity [7,8,15]. Figure 1 shows representative pictures of this analysis.

### Cell count assay

Evaluation of cell proliferation was performed with XTT Reagent kit (XTT, cell proliferation kit, Beit-Ha'Emek, Israel) according to manufactures protocol. This is based on the activity of mitochondria enzymes in live cells, reducing tetrazolium salts, XTT, into colored formazan compounds, which can be detected colorimetric with a spectrophotometer at 450 nm (ELISA reader). Dye absorbance is proportional to the number of cells in each well.

### Substrate-gel-electrophoresis (zymography)

In order to detect proteolytic activity in conditioned media (CM) collected after 48–72 h culture, substrate-gel-electrophoresis (Zymography) on gels containing gelatin as the substrate were used as was described by our previous manuscript [4]. Briefly, CM, was diluted in sample buffer (5% sodium dodecyl sulphate (SDS), 20% glycerol in 0.4 mol/l Tris, pH 6.8 containing 0.02% Bromophenol Blue without 2-mercaptoethanol) and electrophoresed, through a 10% polyacrylamid gel containing 0.5% gelatin (50 mg/ml). Afterwards gels were washed twice in 2.5% Triton X-100 for 15 min. and incubated for 24 h at 37°C in 0.2 mol/l NaCl, 5 mmol/l CaCl_2_, 0.2% Brij 35 and 50 mmol/l Tris, pH 7.5. The buffer was decanted and the gels stained with Coomassie Blue G in 30% methanol and 10% acetic acid for 10 min at room temperature on a rotary shaker. Stain was washed out with water until clear bands were seen. Areas where proteolytic activity degraded the gelatin were seen as absence of staining. Identification of each gelatinase band was done in accordance to their molecular weight and commercial standards (gelatinize A and B, 7 μl; Oncogene Science, Cambridge, MA, data not shown). These bands (proMMP) were quantified using the BioImaging gel documentation system (Dinco & Renum, Jerusalem, Israel) endowed with TINA software (Raytest, Staubenhardt, Germany). MMP secretion was expressed as percent of control.

### Matrigel invasion assay

Matrigel invasion assay was prepared in our laboratory with modifications as described in detail elsewhere [17,18]. Briefly, diluted 1:10 Matrigel (1 mg/ml) (BD Biosciences, Beit-Ha'Emek, Israel) in serum free cell culture media was added to upper chamber of 24-well transwell plate, and incubated at 37°C 3–4 h for gelling. JAR Cells were harvested from tissue culture flasks by Trypsin/EDTA, washed and resuspended in 1.5% FCS in M-199 medium and added to upper wells at a density of 10^5 ^cells/well in 200 μl medium, while 500 μl medium was added to lower well. 1^st ^trimester trophoblast were cultured in upper wells at a density of 2 × 10^5 ^cells/well in 100 μl medium. The same density of cells, in the absence or presence of activators, was seeded in a well without transwell and counted at time of the invasion assay, as reference of total cells. Preliminary studies found no significant matrigel-mediated changes in multiplication rates between 6–8 w and 9–12 w trophoblasts, whether seeded on matrigel or at plastic bottom of well (data not shown). Activators (10 μM Forskolin or 8 ng/ml EGF) and inhibiting MMP-2 or MMP-9 antibodies (Oncogene Cat. IM33L, Cat. IM09L; concentration as recommended by manufacture) were added to medium in upper and lower wells. Plates were incubated at 37°C for 36–48 hours, and then non-invaded cells on top of the transwell were scraped off with a cotton swab. The amount of invaded cells in the lower well as a percent of total seeded cells was evaluated with XTT Reagent kit. The percent of invasion was calculated as:



Invasion was expressed as Invasion Index (Percent of control).

### Statistical methods

Results are expressed as mean ± SEM of 5–10 independent experiments, each treatment performed in duplicates. Statistical analysis was performed using the SPSS statistical software. Student's t-test and "one way analysis of variance" (ANOVA) were used when appropriate. P < 0.05 was considered significant.

### Immunohistochemistry

Immunohistochemistry was performed as previously described [19] using the Histostain-Plus kit (Zymed laboratories Inc., USA). Briefly, cultured cells were fixed with cytospray for 20 min and quenched with 3% hydrogen peroxidase in methanol to eliminate endogenous peroxidase activity. The slides were washed, blocked and incubated at room temperature with primary antibodies (mouse anti-human cytokeratin-7 (1:100, clone OVTL12/30, Biogenics) and mouse anti-human vimentin (1:200, clone V9, Zymed laboratories Inc., USA). Secondary antibody used: Histostain-Plus broad-spectrum biotinolated second antibody (Zymed laboratories Inc., USA). Slides were then developed with a substrate-chromagen solution of aminoethyl carbazole (Zymed laboratories Inc., USA).

## Results

### Relative secretion profile of proMMP-2 and proMMP-9 in 6–8 W, 9–12 W trophoblasts and in JAR cells without treatment

**Figure 2:**
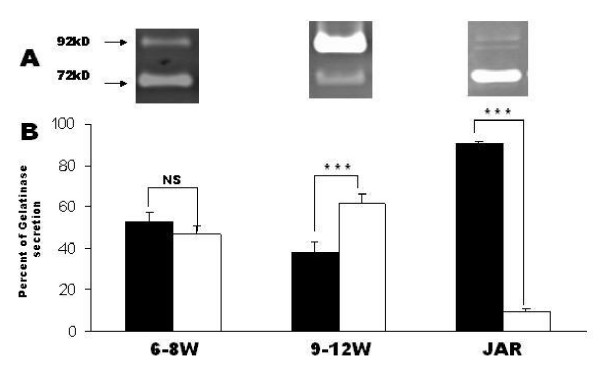
**(A) **Representative secretion pattern of proMMP-2 (72 kD) and proMMP-9 (92 kD) in 6–8 w, 9–12 w trophoblasts and in JAR cells without treatment as examined with zymography. **(B) **Bar graph describing the relative percentage of gelatinases secretion, representing mean ± SEM from 5 independent experiments. Black bars represents proMMP-2 and white bars represent proMMP-9, *P < 0.05.

JAR cells (1 × 10^4 ^cells/well), 1^st ^trimester trophoblast cells 6–8 w and 9–12 w (1–2 × 10^5 ^cells/well) were incubated for 48 hours, then media collected and gelatinase secretion analyzed by zymography. Figure 2 summarizes the results. In 6–8 w trophoblast, proMMP-2 secretion was only slightly dominant (statistically not significant) compared to proMMP-9, 53.2% vs. 46.8% respectively (SEM ± 4.3). In 9–12 w trophoblasts the picture was different, with proMMP-9 being dominant (P < 0.05) over proMMP-2, 61.7% vs.38.3% respectively (SEM ± 4.6). In JAR cells proMMP-2 was dominant (P < 0.05), whereas proMMP-9 only had a small contribution to the gelatinase secretion, 90.2% vs.9.8% respectively (SEM ± 1.4).

### Dose-dependent effect of forskolin and EGF on the proMMP-2 and -9 secretion by JAR cells

**Figure 3:**
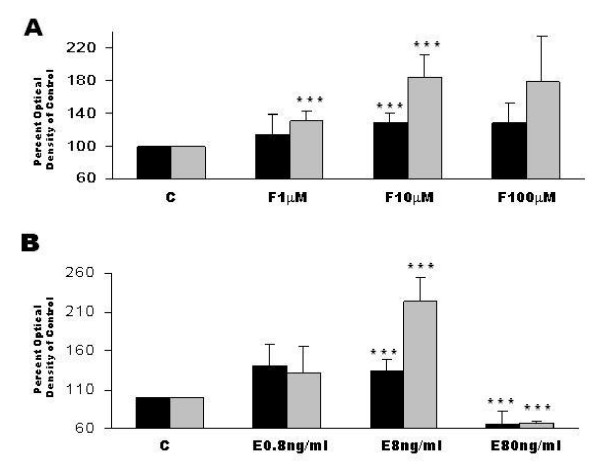
Dose-dependent effect of Forskolin and EGF on gelatinase secretion in JAR cells. JAR cell (1 × 10^4^/well) were incubated 48 hours with or without Forskolin (1 μM, 10 μM or 100 μM) or EGF (0.8 ng/ml, 8 ng/ml or 80 ng/ml) and media collected for measurement of Gelatinase secretion. (**A) **Bar graph, representing mean ± SEM of 10 independent experiments, of cells treated with forskolin. (**B) **Bar graph, representing mean ± SEM of 10 independent experiments, of cells treated with EGF. Black bars represent proMMP-2 and gray bars represent proMMP-9. *P < 0.05 vs. control.

JAR cells (1 × 10^4 ^cells/well) were incubated 48 hours in the absence or presence of forskolin (1 μM, 10 μM or 100 μM) or EGF (0.8 ng/ml, 8 ng/ml or 80 ng/ml) and media was analyzed by zymography for gelatinase secretion. Fig 3 summarizes the results. 10 μM forskolin significantly enhanced secretion of proMMP-2 compared to control (128.5% ± 12.0, P < 0.05). Forskolin (1 μM and 10 μM) significantly enhanced proMMP-9 secretion compared to control (131.3% ± 11.1, and 183.2% ± 27.9, P < 0.05, respectively) (Fig. 3A). 8 ng/ml EGF significantly enhanced secretion of proMMP-2 (133.5% ± 15.0, P < 0.05) and of proMMP-9 (223.9% ± 30.4, P < 0.05) compared to control. 80 ng/ml EGF, on the contrary, decreased proMMP-2 and proMMP-9 secretion compared to control (65.1% ± 18.3, and 66.6% ± 3.7, P < 0.05) (Fig. 3B).

### Effect of forskolin on proMMP-2 and proMMP-9 secretion by JAR cells, 1^st ^trimester trophoblast cells 6–8 w and 9–12 w of gestation

**Figure 4:**
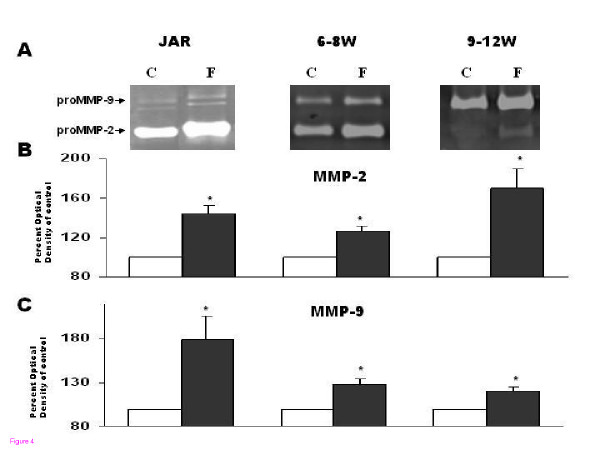
Secretion of proMMP-2 and proMMP-9 after 48 hours incubation of JAR cells, 1^st ^trimester trophoblast cells 6–8 week or 1^st ^trimester trophoblast cells 9–12 week in medium in absence or presence of 10 μM Forskolin. (**A) **Representative zymography gels. (**B) **Bar graph, representing mean ± SEM from 10 independent experiments detecting proMMP-2 (72 kD). (**C**) Bar graph, representing mean ± SEM from 10 independent experiments detecting proMMP-9 (92 kD). White bars represent control medium of cells without treatment. Black bars represent medium from cells with forskolin treatment. *P < 0.05 vs. control.

JAR cells (1 × 10^4 ^cells/well), 1^st ^trimester trophoblast cells 6–8 w and 9–12 w (1–2 × 10^5 ^cells/well) were incubated for 48 hours in the absence or presence of forskolin 10 μM. Figure 4A shows representative zymography gels. Figure 4B and 4C summarizes the results. Gelatinase secretion was enhanced by forskolin in all cell groups: Forskolin significantly increased proMMP-2 secretion in JAR cells (144.3% ± 8.8, P < 0.05), in 6–8 w trophoblast (125.9% ± 6.3, P < 0.05) and in 9–12 w trophoblast (169.7% ± 20.3, P < 0.05) as compared to control (Fig. 4B). Forskolin also significantly increased proMMP-9 secretion in JAR cells (226.6% ± 50.7, P < 0.05), in 6–8 w trophoblast (128.4% ± 6.4, P < 0.05), and in 9–12 w trophoblast (120.3% ± 4.5, P < 0.05) as compared to control (Fig. 4C).

### Effect of EGF on proMMP-2 and proMMP-9 secretion by JAR cells, 1^st ^trimester trophoblast cells 6–8 w and 9–12 w of gestation

**Figure 5:**
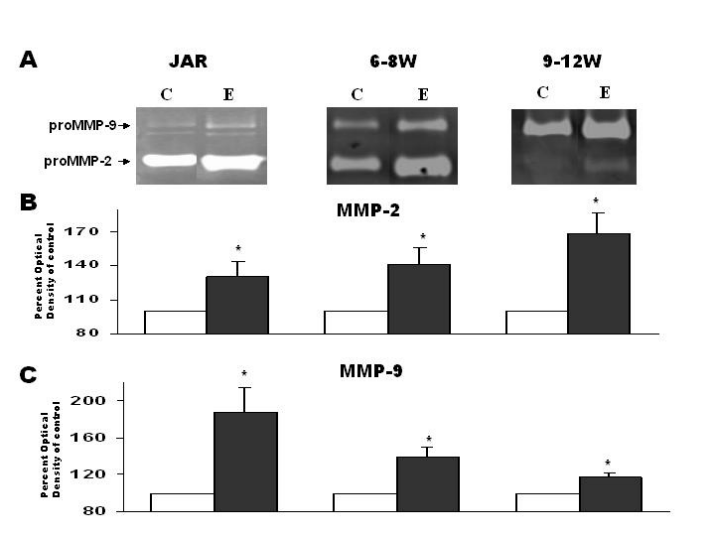
Secretion of proMMP-2 and proMMP-9 after 48 hours incubation of JAR cells, 1^st ^trimester trophoblast cells 6–8 week or 1^st ^trimester trophoblast cells 9–12 week in medium in absence or presence of 8 ng/ml EGF. (**A**) Representative zymography gels. (**B) **Bar graph, representing mean ± SEM from 10 independent experiments detecting proMMP-2 (72 kD). (**C**) Bar graph, representing mean ± SEM from 10 independent experiments detecting proMMP-9 (92 kD). White bars represent control medium of cells without treatment, black bars represent medium from cells with EGF treatment. *P < 0.05 vs. control.

JAR cells (1 × 10^4 ^cells/well), 1^st ^trimester trophoblast cells 6–8 w and 9–12 w (1–2 × 10^5 ^cells/well) were incubated for 48 hours in the absence or presence of EGF 8 ng/ml. Figure 5A shows representative zymography gels. Figure 5B and 5C summarizes the results. EGF significantly increased proMMP-2 secretion in JAR cells (130.4% ± 13.1, p < 0.05), in 6–8 w trophoblast (141.22% ± 14.8, P < 0.05) and also in 9–12 w trophoblast (168.3% ± 18.2, P < 0.05) as compared to control (Fig. 5B). EGF significantly increased proMMP-9 secretion in JAR cells (187.8% ± 27.3, P < 0.05), in 6–8 w trophoblast (138.8% ± 10.3, P < 0.005), and in 9–12 w trophoblast (117.3% ± 3.8, P < 0.05) as compared to control (Fig. 5C).

### Effect of forskolin on cell invasion properties in JAR cells, 1^st ^trimester trophoblast cells 6–8 w and 9–12 w of gestation

**Figure 6:**
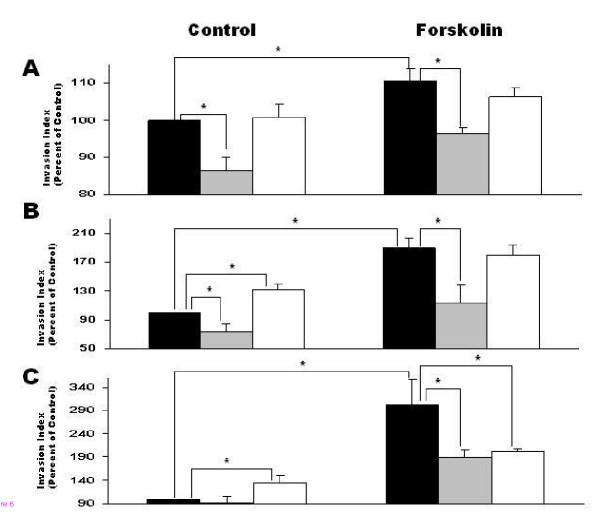
Cell invasion ability of JAR cells, 1^st ^trimester trophoblast cells 6–8 week or 1^st ^trimester trophoblast cells 9–12 week tested with Transwell Invasion Assay. (**A) **represents JAR cells, (**B**) 1^st ^trimester trophoblast cells 6–8 week and (**C**) 1^st ^trimester trophoblast cells 9–12 week. Cells were treated with 10 μM Forskolin and incubated 36 hours on Matrigel coated membrane, with or without MMP-2 or MMP-9 inhibitory antibodies. Cells that invaded the membrane to lower well were counted with XTT. Results represent mean ± SEM from 10 independent experiments. Black bars represent control (cells without treatment or cells treated with Forskolin) with no antibodies. Gray bars represent cells (without treatment or treated with Forskolin) with addition of MMP-2 inhibitory antibody. White bars represent cells (without treatment or treated with Forskolin) with addition of MMP-9 inhibitory antibody. ANOVA for all groups results in p < 0.05, post test confirmed the t test results. *P < 0.05

JAR cells (10^5 ^cells/well), 1^st ^trimester trophoblast cells 6–8 w and 9–12 w (2 × 10^5 ^cells/well) were incubated for 36–48 hours in the absence or presence of forskolin 10 μM on top of Transwell wells containing a transwell membrane coated with matrigel. Forskolin (10 μM) significantly enhanced trophoblast invasion in all cell groups. Forskolin increased cell invasion in JAR cells (110.6% ± 3.4, P < 0.05) (Fig. 6A), in 6–8 w 1^st ^trimester trophoblast (189.7% ± 14.2, P < 0.05) (Fig. 6B) and in 9–12 w (302.4% ± 56.0, P < 0.05) as compared to control (Fig. 6C). The addition of inhibitory MMP-2 antibody significantly decreased invasion of control cells of JAR cells (86.5% ± 3.6, P < 0.05) and of 6–8 w (73.8 ± 10.6, P < 0.05) but did not affect 9–12 w trophoblasts. In forskolin-induced cells the presence of inhibitory MMP-2 antibody caused a significant decrease in invasion of JAR cells compared with induced cells alone (96.6% ± 1.5 versus 110.6% ± 3.4, respectively P < 0.05), of 6–8 w trophoblasts compared with induced cells alone (114.1% ± 24.6 versus 189.7% ± 14.2, respectively, P < 0.05) and of 9–12 w trophoblasts compared with induced cells alone (188.8% ± 18.4 versus 302.4% ± 56.0, respectively, P < 0.05) (Figure. 6A,6B and 6C)

The addition of inhibitory MMP-9 antibody surprisingly increased invasion of control cells of 6–8 w and 9–12 w trophoblast (132.0 ± 8.3 and 134.9 ± 15.2, respectively, P < 0.05) as compared to control, and only in 9–12 w forskolin-induced cells caused a significant decreased invasion compared with induced cells alone (201.5% ± 6.3 versus 302.4% ± 56.0, respectively, P < 0.05). ANOVA post test confirmed the t test results (Fig. 6A,6B and 6C).

### Effect of EGF on cell invasion properties in JAR cells, 1^st ^trimester trophoblast cells 6–8 w and 9–12 w of gestation

**Figure 7:**
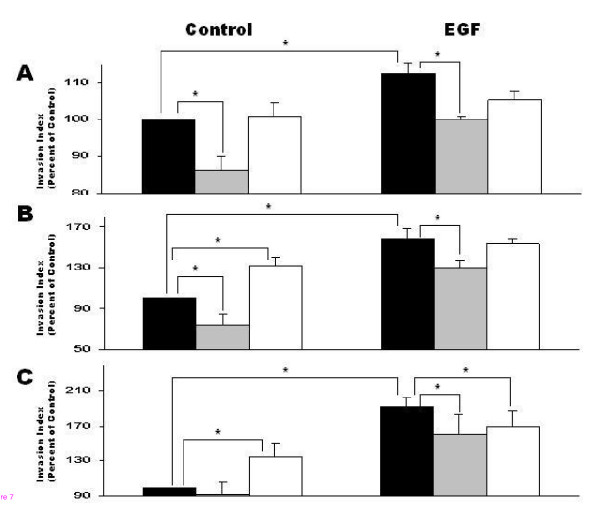
Cell invasion ability of JAR cells, 1^st ^trimester trophoblast cells 6–8 week or 1^st ^trimester trophoblast cells 9–12 week tested with Transwell Invasion Assay. (A) represents JAR cells, (B) 1^st ^trimester trophoblast cells 6–8 week and (C) 1^st ^trimester trophoblast cells 9–12 week. Cells were treated with 8 ng/ml EGF and incubated 36 hours on Matrigel coated membrane, with or without MMP-2 or MMP-9 inhibitory antibodies. Cells that invaded the membrane to lower well were counted with XTT. Results represent mean +SEM from 10 independent experiments. Black bars represent control (cells without treatment or cells treated with EGF) with no antibodies. Gray bars represent cells (without treatment or EGF treated) with addition of MMP-2 inhibitory antibody. White bars represent cells (without treatment or EGF treated) with addition of MMP-9 inhibitory antibody. ANOVA for all groups results in p < 0.05, post test confirmed the t test results. * P < 0.05.

JAR cells (10^5 ^cells/well), 1^st ^trimester trophoblast cells 6–8 w and 9–12 w (2 × 10^5 ^cells/well) were incubated for 36–48 hours in the absence or presence of EGF 8 ng/ml on top of Transwell wells containing a transwell membrane coated with matrigel. The results of EGF resembled those of forskolin. EGF enhanced trophoblast invasion in all cell groups. EGF increased cell invasion in JAR cells (112.6% ± 2.9, P < 0.05) (Fig. 7A), in 6–8 w 1^st ^trimester trophoblast (157.9% ± 10.4, P < 0.05) (Fig. 7B) and in 9–12 w (192.4% ± 10.5, P < 0.05) compared to control (Fig. 7C). In EGF-induced cells the presence of inhibitory MMP-2 antibody caused a significant decrease in invasion of JAR cells (100.2% ± 0.8 versus 112.6 ± 2.9, P < 0.05), of 6–8 w trophoblasts (129.7% ± 8.0 versus 157.9 ± 10.4, P < 0.05) and of 9–12 w trophoblasts (161.1% ± 22.0 versus 192.4 ± 10.5, P < 0.05) compared with induced cells alone (Figure. 7A,7B and 7C).

The addition of inhibitory MMP-9 antibody affected 9–12 w EGF–induced cells and caused a significant decreased invasion compared with induced cells alone (169.7% ± 18.1 versus 192.4 ± 10.5, respectively, P < 0.05), ANOVA post test confirmed the t test results (Figure. 7A,7B and 7C).

The inhibitory affect of MMP-2/-9 antibody on control cells was described in the previous section.

## Discussion

Trophoblastic invasion of the endometrium is highly regulated by interrelated reactions between invasion-promoting factors, such as cytokines, growth factors, MMP-2 and MMP-9, and invasion-inhibiting factors such as TIMPs.

In the current study, the secretion and activity of MMP-2 and MMP-9 in human cytotrophoblastic cells from different weeks of gestation was measured and compared with a choriocarcinoma cell-line.

A differential secretion profile of proMMP-2 and -9 was found between 6–8 w and 9–12 w as expressed by a shift in the relative proportion of each gelatinase. Elevated proMMP-9 secretion in 9–12 w was observed, compared with both 6–8 w and JAR cells. These results are consistent with those of Xu et al [11], who found MMP-2 production to be dominant between 6–8 weeks of gestation and then declining, whereas MMP-9 production significantly increased from 8 to 11 weeks, with a shift of dominant gelatinase from MMP-2 to MMP-9 from 9 week of gestation. MMP-2 is predominant in human preimplantation embryos [20,21], whereas MMP-9 is dominant in the third trimester [6]. Niu et al [22] reported a dominance of MMP-2 secretion over MMP-9 from 1^st ^trimester villous tissue, and a dramatically decrease in MMP-2 levels in the second trimester. These results support our findings of a dynamic gelatinase secretion profile during the 1^st ^trimester.

In our study, MMP secretion was induced by two separate signal pathways: PKA and PTK. Several factors with importance in embryo implantation act via the cAMP-protein kinase A (PKA) signal transduction pathway, including human chorion gonadotropin (hCG), the primary signal of an implanting pregnancy [23]. Forskolin is a prototypical stimulator of the cAMP pathway by direct activation of adenylate cyclase [24].

In this study, forskolin was found to significantly enhance proMMP-2 secretion in JAR choriocarcinoma cell-line, 6–8 w and 9–12 w trophoblasts. Forskolin also enhanced proMMP-9 secretion, in choriocarcinoma cells and in 1^st ^trimester trophoblast (from both groups). This indicates that forskolin might influence trophoblast cells invasiveness by enhancing the secretion of gelatinases. Zymography measures all forms of MMP (active and inactive) and therefore does not reliably represent the true physiological activity, which is influenced by the presence of MMP inhibitors and activators. In order to examine the role of the gelatinases in the implantation process, we examined cell invasion after PKA stimulation in JAR cells, 6–8 and 9–12 w trophoblasts. Our results showed a significant increase in invasive ability in JAR cells and in early and late 1^st ^trimester trophoblasts, after stimulation with forskolin. In order to detect the contribution of each gelatinase to this invasive process, inhibitory antibodies to MMP-2 and MMP-9 were added to cell culture and cell invasive ability examined. MMP-2 inhibitory antibody caused a significant decrease in cell invasion in JAR cells, 6–8 w and 9–12 w trophoblasts treated with forskolin, whereas MMP-9 inhibitory antibody only caused a decrease in 9–12 w trophoblasts. This indicates, that most probably MMP-2 and not MMP-9 is the key-enzyme in the invasion process of JAR cells and early 1^st ^trimester trophoblasts stimulated by forskolin, whereas MMP-9 together with MMP-2 plays a role in late 1^st ^trimester trophoblasts. Only a few published data describe the relationship between forskolin and trophoblast invasion. Human chorionic gonadotropin acts via cAMP, and is considered a sign of differentiation of trophoblasts to syncytiotrophoblast. The hCG receptor was shown to be expressed on invasive trophoblast and in choriocarcinoma cells, and hCG was found to increase *in vitro *invasion and migration of a trophoblastic cell line, an effect that was also mimicked by forskolin [23]. Several studies demonstrated a positive correlation between hCG level and successful implantation [25] or inappropriate implantation/ invasion associated with the development of preeclampsia [24] or gestational trophoblastic tumors [27]. To the best of our knowledge, our study is the first to report the effect of forskolin on MMPs in first trimester trophoblastic cells. In addition, this study also distinguishes between the contributions of each of the gelatinases to the invasive capacity enhanced by forskolin in trophoblastic cells.

Epidermal Growth factor (EGF) plays a major role in placental implantation, growth and differentiation and is regarded a paracrinic factor modifying the implantation process. EGF acts on trophoblasts via a specific receptor (EGFR) from the tyrosine kinase receptor family [28]. EGF is secreted from the endometrium during the implantation window, in which the embryo also expresses EGFR [29], and expressed in placenta from 1^st ^throughout third trimester [30]

In this study EGF stimulated secretion of proMMP-2 in JAR cells, 6–8 w and 9–12 w trophoblasts and also enhanced the secretion of proMMP-9 in JAR, in 6–8 w trophoblasts and in 9–12 w trophoblasts. Our results therefore indicate, that MMP-2 and MMP-9 secretion by trophoblastic cells may be stimulated through the PTK pathways during the first trimester. We noted that EGF at a high concentration (80 ng/ml), in contrast, decreased proMMP-2 and -9 secretion in JAR cells. This result corresponds with previous published data regarding a dual, concentration dependent effect of EGF on cell functions [31,32].

In order to examine the role of the gelatinases in the implantation process, we examined cell invasion after PTK stimulation in JAR cells, 6–8 and 9–12 w trophoblasts. Our results showed a significant increase in invasion ability in JAR cells and in early and late 1^st ^trimester trophoblasts, after stimulation with EGF. In EGF-stimulated JAR cells and in 6–8 w and 9–12 w trophoblasts inhibitory MMP-2 antibody decreased cell invasion, whereas inhibitory MMP-9 antibody caused a significant decrease in invasion only in 9–12 w trophoblasts. We thereby showed, that in EGF stimulated cells as well, MMP-2 is the key-enzyme in the invasion process *in vitro *in JAR cell and in early 1^st ^trimester trophoblasts, whereas in late 1^st ^trimester trophoblasts both MMP-2 and MMP-9 have a role. EGF was found to induce changes in morphology and to increase invasive capacity of first trimester trophoblasts, whereas later gestational cytotrophoblasts (2^nd ^trimester), whose invasive capacity is diminished, are much less affected [29]. EGF was also found to increase MMP-9 secretion by cytotrophoblasts [33].

In JAR control cells inhibitory, MMP-2 antibody also decreased invasion, whereas MMP-9 antibody had no affect, indicating that the basic invasive ability of these choriocarcinoma cells is mainly due to MMP-2 and not to MMP-9. Inhibition of MMP-2 in control cells also decreased invasion in 6–8 w trophoblast, but not in 9–12 w, indicating again the importance of MMP-2 in early (6–8 w) trophoblast invasiveness. Surprisingly inhibition of MMP-9 in 6–8 w and 9–12 w trophoblasts without treatment caused an increase in invasion. We speculate that this may be due to a release of other proteinases, including other MMPs, since MMP-9 is known to dimerize [34,35] All in all we found, that in early 1^st ^trimester trophoblasts (6–8 w), MMP-2 is the major gelatinase participant in cell invasion, whereas in later 1^st ^trimester trophoblasts (>9 w) both MMP-9 and MMP-2 most probably participate in cell invasion, however we cannot exclude the possibility of other MMP family members participating in this process. Isaka et al [9] have shown that invasive ability of early first trimester trophoblast was inhibited by MMP-2 antibody in a dose dependent manner, thereby suggesting that the invasive ability of trophoblasts may be regulated by the enzyme activity of gelatinases, especially MMP-2. This study supports ours in the involvement of MMP-2 in trophoblast cell invasion. MMP-2 was found to be located in invasive evCTB in 1^st ^trimester placenta [9,10,36], whereas MMP-9 was located in the non-invasive vCTB [9]. In contrast, several studies have found MMP-9 to be the key-enzyme in trophoblast invasion *in vitro *[1,5,7]. We speculate, that the main reason for this controversy of results comes from the dynamic gelatinase expression during the 1^st ^trimester, as earlier discussed. The choice of pathway for stimulation of cell invasion may also contribute to a difference in results. We used stimulation through the PKA and PTK pathways; whereas other groups used the PKC pathway [7,8]. Various stimulators, inducing different signal pathways, are likely not to affect the same enzymes in an identical manner, and thereby can result in varying dominant enzymes.

We found, that MMP-2 is also the key-enzyme in JAR cell invasion; therefore JAR cells resemble early 1^st ^trimester trophoblasts in cell invasive ability and in MMP secretion profile and differ from late 1^st ^trimester trophoblast in these parameters.

It has been documented, that JAR-trophoblast cells have the ability to invade in vivo [37,38]. We chose this cell-line because it provides a large number of uniform cells and preserve the ability to differentiate into syncytiotrophoblast-like cell in vitro [39,40]. Other studies showed different compartment in vitro between choriocarcinoma cell-lines and human first trimester trophoblast in the regulation of invasion [36,37]. The study of JAR cell invasion may therefore represent only partly the aspects and mechanisms of the in vivo situation of invasion, where many cell types are involved.

## Conclusions

We showed that forskolin and EGF stimulate proMMP-2 and -9 secretion from trophoblasts, and that there is a differential, dynamic importance of each gelatinase in trophoblast invasion during 1^st ^trimester. We suggest that MMP-2 is the key-enzyme in JAR and early 1^st ^trimester (6–8 w) trophoblast invasion, whereas both MMP-2 and -9 are important for late (9–12 w) trophoblast invasion.
